# Metagenomic Analysis of RNA Fraction Reveals the Diversity of Swine Oral Virome on South African Backyard Swine Farms in the uMgungundlovu District of KwaZulu-Natal Province

**DOI:** 10.3390/pathogens11080927

**Published:** 2022-08-17

**Authors:** Ravendra P. Chauhan, James E. San, Michelle L. Gordon

**Affiliations:** 1 School of Laboratory Medicine and Medical Sciences, College of Health Sciences, University of KwaZulu-Natal, Durban 4001, South Africa; 2 KwaZulu-Natal Research Innovation and Sequencing Platform (KRISP), School of Laboratory Medicine and Medical Sciences, University of KwaZulu-Natal, Durban 4001, South Africa; 3 Center for Epidemic Response and Innovation (CERI), School of Data Science and Computational Thinking, Stellenbosch University, Stellenbosch 7600, South Africa

**Keywords:** backyard swine, deep sequencing, Illumina sequencing, phylogenetic analysis, RNA viruses, South African backyard farms, swine oral virome, swine viruses, zoonosis

## Abstract

Numerous RNA viruses have been reported in backyard swine populations in various countries. In the absence of active disease surveillance, a persistent knowledge gap exists on the diversity of RNA viruses in South African backyard swine populations. This is the first study investigating the diversity of oral RNA virome of the backyard swine in South Africa. We used three samples of backyard swine oral secretion (saliva) collected from three distantly located backyard swine farms (BSFs) in the uMgungundlovu District, KwaZulu-Natal, South Africa. Total viral RNA was extracted and used for the library preparation for deep sequencing using the Illumina HiSeq X instrument. The FASTQ files containing paired-end reads were analyzed using Genome Detective v 1.135. The assembled nucleotide sequences were analyzed using the PhyML phylogenetic tree. The genome sequence analysis identified a high diversity of swine enteric viruses in the saliva samples obtained from BSF2 and BSF3, while only a few viruses were identified in the saliva obtained from BSF1. The swine enteric viruses belonged to various animal virus families; however, two fungal viruses, four plant viruses, and five unclassified RNA viruses were also identified. Specifically, viruses of the family *Astroviridae*, according to the number of reads, were the most prevalent. Of note, the genome sequences of Rotavirus A (RVA) and Rotavirus C (RVC) at BSF2 and RVC and Hepatitis E virus (HEV) at BSF3 were also obtained. The occurrence of various swine enteric viruses in swine saliva suggests a high risk of diarrhoeic diseases in the backyard swine. Of note, zoonotic viruses in swine saliva, such as RVA, RVC, and HEV, indicate a risk of zoonotic spillover to the exposed human populations. We recommend the implementation of biosecurity to ensure sustainable backyard swine farming while safeguarding public health.

## 1. Introduction

Backyard swine (*Sus scrofa domesticus*) farming supports the rural economy and simultaneously ensures food security in rural and semi-urban South African communities [1]. Lack of knowledge, resources, and inadequate biosecurity at the backyard farms are the known factors that pose significant risks that might facilitate virus disease outbreaks endangering backyard swine farming and public safety [2,3,4,5]. The emergence of the 2009 influenza pandemic in the Mexican swine [3,6], caused by influenza A virus (IAV) [7,8], is an example of how zoonotic viruses may evolve and trigger a pandemic. Apart from the IAV [5,8,9], several other viral pathogens may also transmit from swine to humans, causing disease outbreaks. For example, the zoonotic transmission of the Nipah virus (NiV) [9] from the swine to humans at swine farms in Malaysia resulted in the death of 105 swine farm workers during 1998–1999 [10,11]. The occurrence of NiV antibodies in swine populations in Australia [12], Bangladesh [13], Ghana [14], and more recently in Uganda in 2019 [15] suggested a continued circulation and, therefore, a constantly growing risk of NiV disease outbreak. 

Numerous other notifiable RNA viruses, including Porcine epidemic diarrhoea virus (PEDV), which is an emerging porcine coronavirus [16,17,18] and causes vomiting and diarrhoea in neonatal piglets, has been reported to inflict 90–100% piglet mortality, leading to substantial economic losses to the swine farmers in many countries [16,19]. Interestingly, PEDV was identified as the cause of death for over eight million neonatal piglets in a single year after its emergence in the United States in 2013 [19]. In addition, the Classical swine fever virus (CSFV) [20,21], Porcine reproductive and respiratory syndrome virus (PRRSV) [22,23,24], Foot and mouth disease virus (FMDV) [25], and Swine vesicular disease virus (SVDV) [26,27] are other controlled or notifiable RNA viruses which have caused severe diseases in swine populations in various countries in the recent past. 

Furthermore, some of the swine enteric viruses, such as Rotavirus A (RVA), Rotavirus B (RVB), and Rotavirus C (RVC), may cause diarrhoea, weight loss, and mortality in piglets [28]. Notably, rotaviruses may be zoonotically transmitted to the exposed human populations and cause diarrhoea in children [29,30,31,32]. In recent years, several Rotavirus types have been reported in swine populations in many countries [31,33,34,35]. 

In South Africa, Rotavirus infection in piglets was first reported in 1977 [36]. Later, a pilot study in 1993 reported the year-round occurrence of RVA in diarrheic piglets at a swine farm located in Northern Transvaal [37]. Subsequently, the RVA, RVB, and RVC were reported in swine populations from various South African provinces in 1996 [38]. Apart from that, FMDV-associated mortality in commercial swine was first reported in September 2000 in the Camperdown District of the KwaZulu-Natal province [39]. The first reported CSFV disease outbreak occurred in South African swine in June 2005 at a commercial piggery in the Western Cape province and reported a high mortality rate [40]; the CSFV outbreak led to the culling of over 335,000 pigs, resulting in a significant production loss to the swine industry in South Africa [41]. Recently, the prevalence of Hepatitis E virus (HEV) was reported in commercial and communal swine farms located in the Chris Hani and Amathole District Municipalities of the Eastern Cape province [42]. 

It is noteworthy that while the above virus pathogens were reported from the commercial swine populations in South Africa; to our knowledge, there is no study of viruses in backyard swine populations in the country. Since some of these virus pathogens may inflict severe disease outbreaks in swine and can also be zoonotically transmitted to the exposed humans, unravelling their prevalence and diversity is crucial to mitigate the risk of disease outbreaks in resource-scarce rural communities. Therefore, we conducted a study to determine the diversity of the swine oral RNA virome at three distantly located backyard swine farms (BSFs) in the uMgungundlovu District of the KwaZulu-Natal province of South Africa. This study would serve as a reference for the stakeholders to evaluate the disease outbreak risk at the backyard swine farms in South Africa and would assist them in preparing a strategy to mitigate interspecies virus transmission to ensure public safety and sustainable backyard swine farming. 

## 2. Results

**Figure 1:**
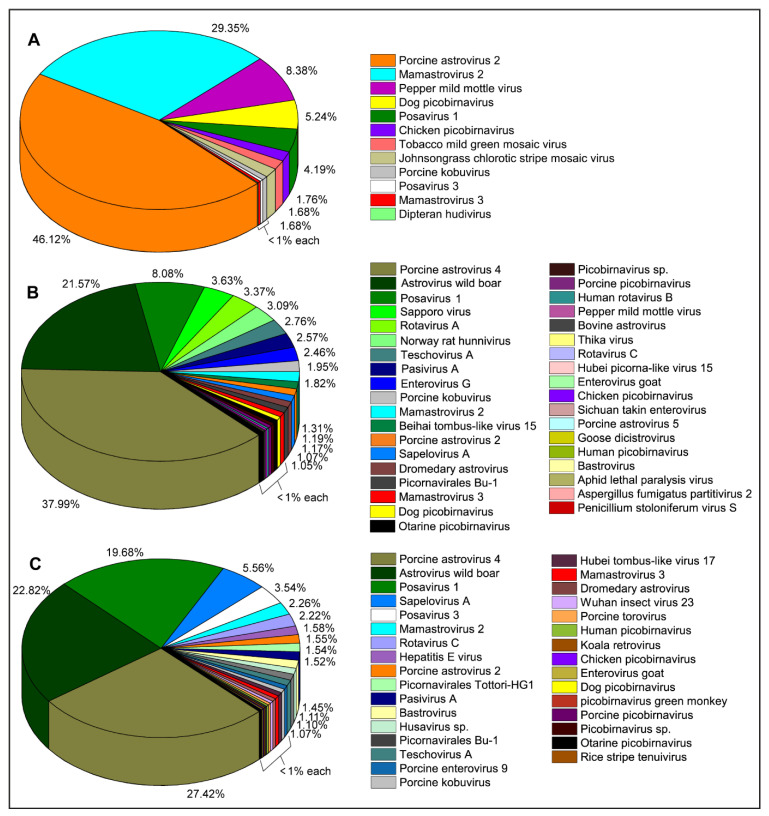
Prevalence (number of raw sequence reads) of RNA viruses in backyard swine saliva samples. Viruses of the family *Astroviridae* were prevalent in the backyard swine saliva samples. (**A**) A higher prevalence of Porcine astrovirus type-2 (PAstV-2) and Mamastrovirus type-2 was detected in a healthy adult swine at BSF1. (**B**) There was a higher prevalence of Porcine astrovirus type-4 (PAstV-4) in piglets with clinical signs of coughing and sneezing at BSF2. (**C**) A higher prevalence of PAstV-4 was identified in grower pigs with red patches on the skin at BSF3. A higher diversity of RNA viruses was determined in saliva obtained from piglets and growers with clinical signs of disease at BSF2 and BSF3, while only a few RNA viruses were identified in a saliva sample obtained from an apparently healthy one-year-old adult swine at BSF1.

The RNA fraction of the backyard swine saliva samples (*n* = 3) generated either near full-length (Figure 1; Table 1) or partial genomes (Supplementary Table S1) which mostly belonged to swine enteric viruses; however, other virus species, such as HEV, two species of fungal viruses, four species of plant viruses, and five species of unclassified RNA viruses were also identified. 

### 2.1. Identification and Characterization of Swine Viruses

#### 2.1.1. Family Astroviridae

**Figure 2:**
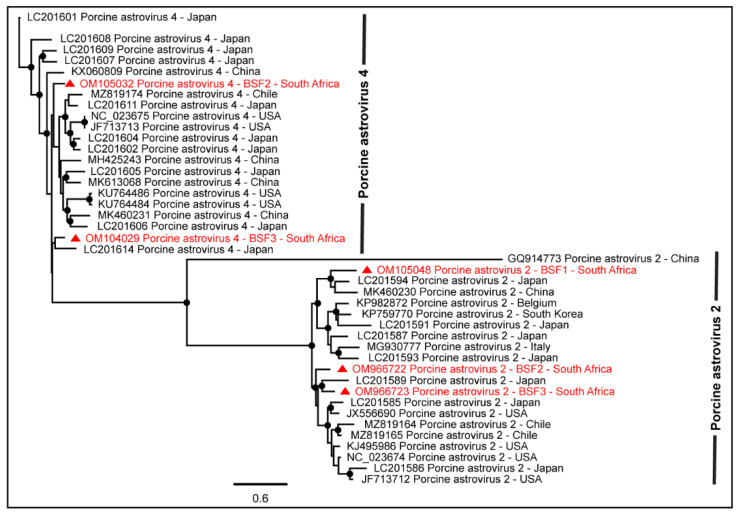
The PhyML phylogenetic tree revealed that PAstV-2 genomes generated from South African backyard swine at BSF1 and BSF3 and PAstV-4 genome generated from BSF3 were closely related to Asian sequences reported from Japan and China. The PAstV-2 and PAstV-4 genomes generated from BSF2 clustered with the sequences reported from Asia and Americas. The black dots at nodes represent ≥80% bootstrap support.

The sequence reads of the viruses in the family *Astroviridae* were prevalent in all three backyard swine saliva samples. While Porcine astrovirus type-2 (PAstV-2) generated the maximum number of reads (46.12%) in a saliva sample obtained from BSF1, the reads of Porcine astrovirus type-4 (PAstV-4) were prevalent in two other saliva samples obtained from BSF2 (37.99%) and BSF3 (27.42%). Two contigs of PAstV-2 were generated from BSF1, one of which consisted of a complete coding sequence (99.3% genome coverage; 6364 nt). In addition, complete coding sequences of PAstV-2 were also generated in BSF2 (99.3% genome coverage; 6313 nt) and BSF3 (96.3% genome coverage; 6127 nt). Complete coding sequences for ORF1ab and ORF1a and partial coding sequence of ORF2 of the PAstV-4 were generated from BSF2 (96.2% genome coverage; 6384 nt) and BSF3 (77% genome coverage; 5111 nt). Interestingly, the PAstV-4 was not identified at BSF1. While the PhyML phylogenetic tree clustered the PAstV-2 sequences obtained from BSF2 and BSF3 with the sequences reported from Japan and Americas, the PAstV-2 sequences generated from BSF1 clustered with Asian sequences reported from Japan and China (Figure 2). 

As seen in Figure 2, the genome sequence of PAstV-4 obtained from the South African BSF3 appeared to be more closely related to the PAstV-4 sequence reported from Japan. Interestingly, the PAstV-4 genome generated from BSF2 was not related to the PAstV-4 genome generated from BSF3 and therefore suggested different origins of the PAstV-4 genomes generated in the present study. Similarly, PAstV-2 genome sequences generated from BSF1, BSF2, and BSF3 did not appear to be related to each other and thus suggested different origins of these sequences in the backyard swine under investigation. In the absence of PAstV-2 and PAstV-4 genome sequences from South Africa and several other geographical locations, the epidemiology of these viruses in backyard swine under investigation remains yet to be determined.

**Figure 3:**
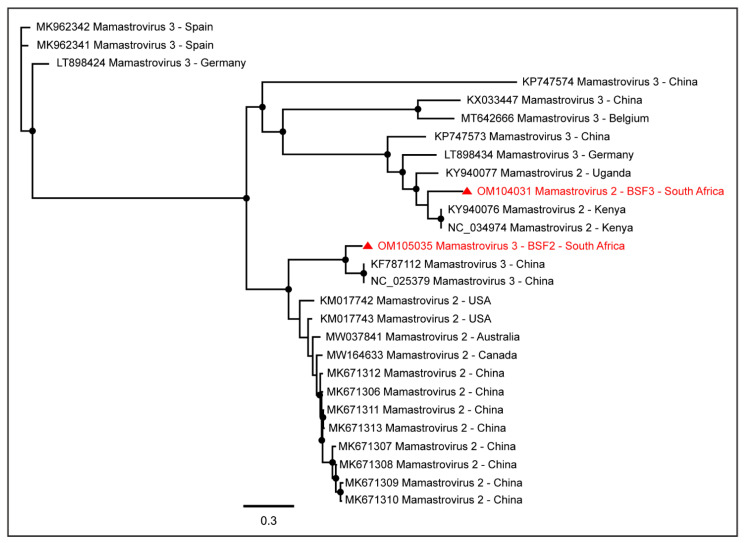
The PhyML phylogenetic tree of Mamastroviruses determined that Mamastrovirus 2 sequences obtained from BSF3 were more closely related to the sequences reported from swine in Kenya, while Mamastrovirus 3 sequences obtained from BSF2 were more closely related to the Chinese sequences. The black dots at nodes represent ≥80% bootstrap support.

While a complete coding sequence of Mamastrovirus 3 (97.9% genome coverage; 6562 nt) was generated from BSF2, partial sequences of Mamastrovirus 2 were generated from BSF3 (84.1% genome coverage; 5338 nt), BSF2 (77.5% genome coverage; 4920 nt), and BSF1 (54.1% genome coverage; 3401 nt). While Mamastrovirus 3 sequences obtained from BSF2 appeared to be more closely related to the Chinese sequences, the Mamastrovirus 2 sequences obtained from the BSF3 appeared to be more closely related to the African sequences reported from swine in Kenya (Figure 3). 

**Figure 4:**
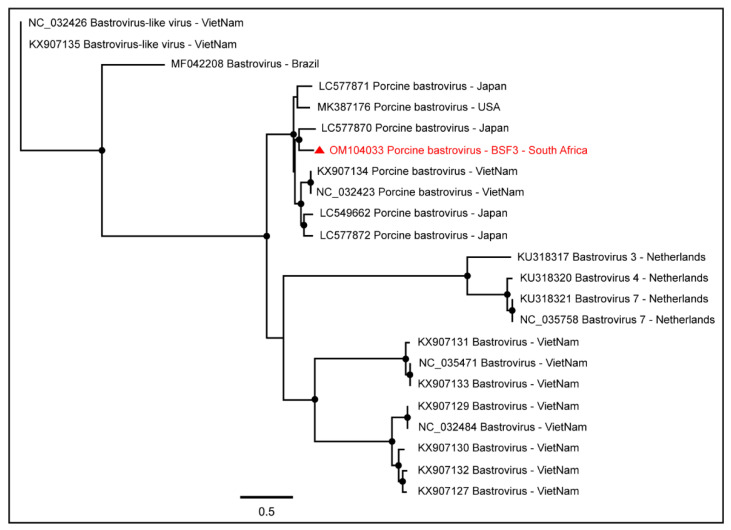
The PhyML phylogenetic tree of Porcine bastroviruses determined that South African Porcine bastrovirus sequences generated from the backyard swine saliva at BSF3 clustered with the Japanese Porcine bastrovirus sequence. The black dots at nodes represent ≥80% bootstrap support.

One complete coding sequence of Porcine bastrovirus (97.4% genome coverage; 5848 nt) was obtained from BSF3. The South African genome of Porcine bastrovirus appeared to be closely related to the Japanese Porcine bastrovirus genome (Figure 4).

The complete coding sequences for ORF1ab and ORF1a and partial coding sequence for ORF2 of Astrovirus wild boar/WBAstV-1/2011/HUN were generated from BSF3 (87.0% genome coverage; 5833 nt). In addition, the complete coding sequence for ORF1a and the partial coding sequence for ORF2 of Astrovirus wild boar/WBAstV-1/2011/HUN were generated from BSF2 (92.7% genome coverage; 6219 nt). They shared only 89.4% pairwise sequence identity with each other and therefore might have different origins. Partial genome sequences of Dromedary astrovirus (89.7% genome coverage; 5674 nt) and Bovine astrovirus (29.3% genome coverage; 1829 nt) were generated from BSF2 and a Dromedary astrovirus genome was generated from BSF3 (47.1% genome coverage; 2979 nt).

#### 2.1.2. Family Hepeviridae

**Figure 5:**
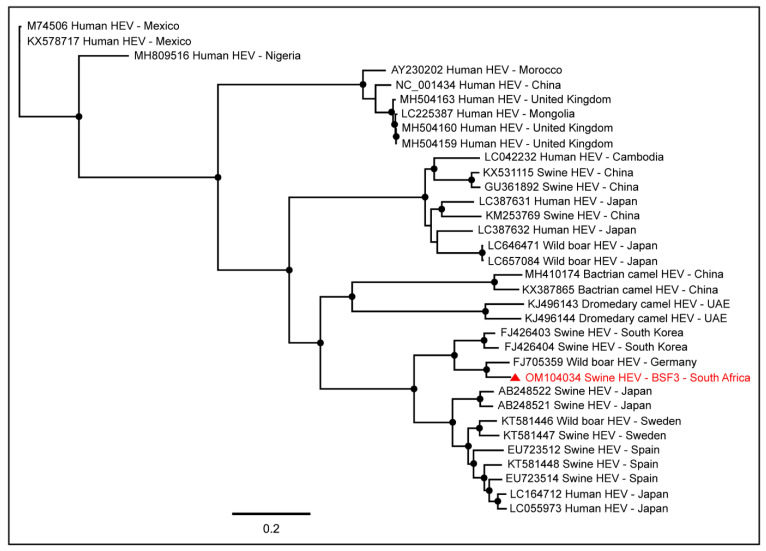
The PhyML phylogenetic analysis of the HEV genome generated from the backyard swine at BSF3 in South Africa determined that this genome was closely related to an HEV genome reported from a wild boar in Germany. The black dots at nodes represent ≥80% bootstrap support.

A near full-length genome (97.7% genome coverage; 7040 nt) of Hepatitis E virus (HEV; *Orthohepevirus A*) was obtained from BSF3. This genome had a complete coding sequence for hypothetical protein and capsid protein and a partial coding sequence for the nonstructural protein. To determine the epidemiology of the South African swine HEV genome, we included in the phylogenetic tree the full-length HEV genome sequences available in the NCBI-GenBank reported from various hosts, including humans, swine, wild boars, bactrian camels, and dromedary camels, from different geographical locations. Interestingly, the South African swine HEV genome generated from the backyard swine at BSF3 in the present study clustered with the HEV genome reported from a wild boar in Germany (Figure 5). However, previously in South Africa, Adelabu et al. (2017) detected HEV RNA in swine fecal samples on the commercial and communal swine farms in the Eastern Cape province, they only generated partial capsid protein sequences ranging from 388–427 nucleotides (GenBank accessions: KX896664–KX896670) [42] which were not included in the PhyML tree in the present study. In the absence of the other full-length swine HEV genome sequences from South Africa, the epidemiology of the swine HEV genome generated in this study remains inconclusive.

#### 2.1.3. Family Picornaviridae

**Figure 6:**
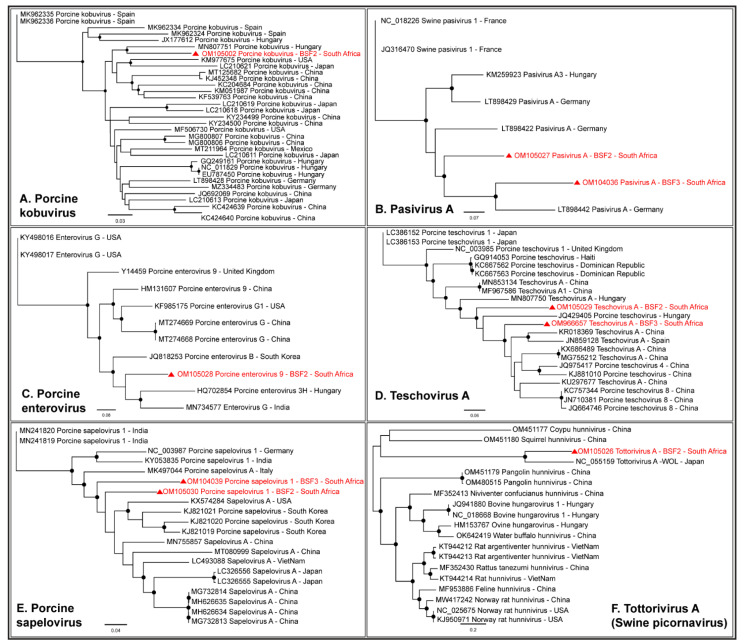
The PhyML phylogenetic analysis of viruses of the family *Picornaviridae*. (**A**) South African sequences of Porcine kobuvirus appeared to be related to the sequences reported from Hungary. (**B**) South African sequences of Pasivirus A appeared to be closely related to the sequences reported from Germany. (**C**) South African Porcine enterovirus 9 genome was related to the sequences reported from Asia and Europe. (**D**) Porcine teschovirus A sequences obtained from BSF2 and BSF3 clustered into different groups suggesting different origins. (**E**) South African sequences of Porcine sapelovirus 1 appeared to be somewhat related to each other. (**F**) A partial genome sequence that was generated from BSF2 grouped with a novel swine picornavirus, Tottorivirus A, with 100% bootstrap support. Tottorivirus A genome appears to have diverged from the hunnivirus genome. The black dots at nodes represent ≥80% bootstrap support.

A complete coding sequence of Porcine kobuvirus (98.9% genome coverage; 8123 nt) was obtained from the South African backyard swine at BSF2, which appeared to be closely related to the Kobuvirus genome reported from Hungary (Figure 6A). In addition, partial coding sequences of Porcine kobuvirus (25.8–54.8% genome coverage) were generated from the BSF1 and BSF3. While one complete coding sequence of Pasivirus A (99.4% genome coverage; 6876 nt) was generated from BSF2, a near-complete coding sequence of Pasivirus A (96.4% genome coverage; 6672 nt) was generated from BSF3 (Figure 6B). A complete coding sequence of Porcine enterovirus 9 (99.4% genome coverage; 7351 nt) was obtained from BSF2 (Figure 6C). In addition, a partial coding sequence of Porcine enterovirus 9 (87.8% genome coverage; 6485 nt) was obtained from BSF3. Two partial coding sequences of Enterovirus goat (31.6% genome coverage; 2354 nt and 37.2% genome coverage; 2779 nt) and Sichuan takin enterovirus (32.3% genome coverage; 2104 nt and 43.5% genome coverage; 2838 nt) were generated from BSF2 and BSF3. While one complete coding sequence of Porcine teschovirus A (98.3% genome coverage; 7001 nt) was generated from BSF2, another complete coding sequence (99.4% genome coverage; 7078 nt) was obtained from BSF3 (Figure 6D). Two complete coding sequences of Porcine sapelovirus 1 (99.7% genome coverage; 7473 nt and 99.3% genome coverage; 7438 nt) were generated from BSF2 and BSF3, respectively (Figure 6E). One partial coding sequence (95.5% genome coverage; 6746 nt) which we initially identified as the Norway rat hunnivirus appears to be more closely related to a novel swine picornavirus named Tottorivirus A, with 100% bootstrap support, after a reference genome recently became available in the NCBI-GenBank (accession: NC_055159), reported from swine feces in Japan. Using PhyML phylogenetic analysis, it appears that the Tottorivirus A might have evolved from the hunnivirus genome (Figure 6F). It should be noted that only a few genome sequences of these picornaviruses were available from swine or other mammals and originated from limited geographical locations. Due to the existing gap in the genome sequence information, the phylogenetic analysis could not determine the epidemiology of the South African swine picornavirus genomes generated in this study for which more genome sequences from diverse geographical locations are needed.

#### 2.1.4. Order Picornavirales

**Figure 7:**
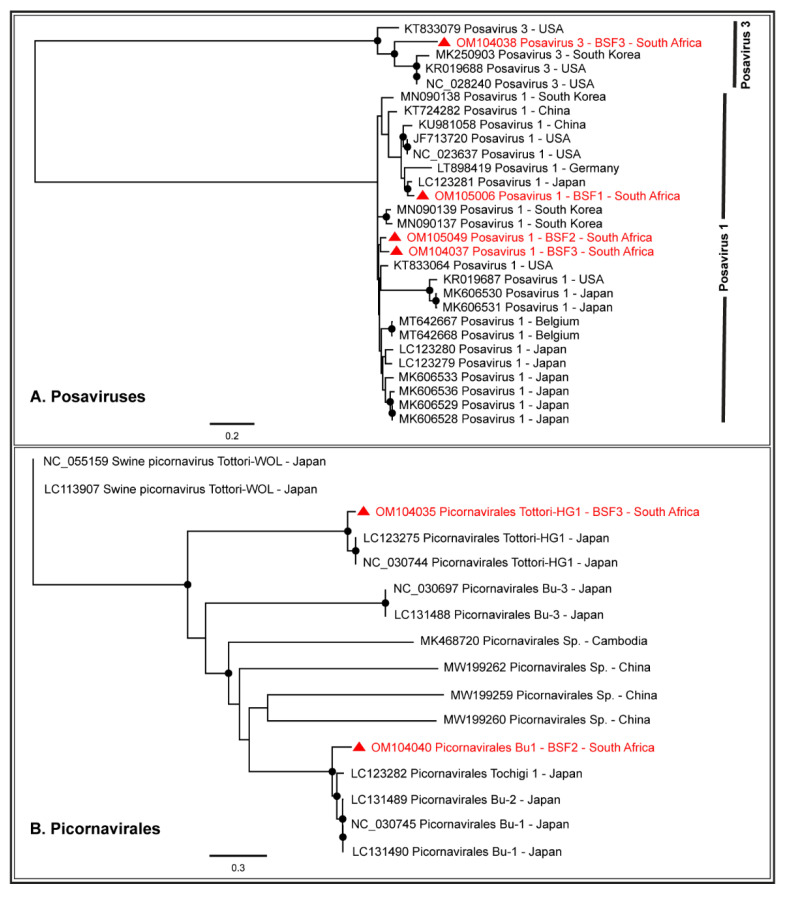
The PhyML phylogenetic analysis of Picornavirales. (**A**) Posavirus 1 genomes generated from the South African backyard swine at BSF2 and BSF3 were related, however, the one generated from BSF1 was related to the genome reported from Japan. Posavirus 3 genome generated from South African backyard swine was related to the genomes reported from South Korea and the USA. (**B**) Picornavirales Bu-1 and Picornavirales Tottori-HG1 genomes generated from the South African backyard swine appeared to be related to the genomes reported from Japan. The black dots at nodes represent ≥80% bootstrap support.

Either partial or complete coding sequences of Posavirus 1 (96.1–99.3% genome coverage) and Posavirus 3 (21.4–99.6% genome coverage) were obtained from the three BSF. While the South African Posavirus 1 sequences obtained from BSF2 and BSF3 appeared to be related to each other, the Posavirus 1 sequences obtained from BSF1 appeared to be related to the Japanese sequences. The South African Posavirus 3 sequences generated from BSF3 appeared to be related to the genomes reported from South Korea and the USA (Figure 7A). One complete coding sequence of Picornavirales Tottori-HG1 (99.2% genome coverage; 9787 nt) and the partial coding sequence of Picornavirales Bu-1 (96.2% genome coverage; 8877 nt) were generated from BSF3 and BSF2, respectively. The Picornavirales Bu-1 and Picornavirales Tottori-HG1 sequences obtained from the South African backyard swine appeared to be related to the genome sequences reported from Japan (Figure 7B). Only a limited number of full-length genome sequences of Picornavirales Tottori HG-1 and Picornavirales Bu-1 were available in the NCBI-GenBank.

#### 2.1.5. Family Reoviridae

**Figure 8:**
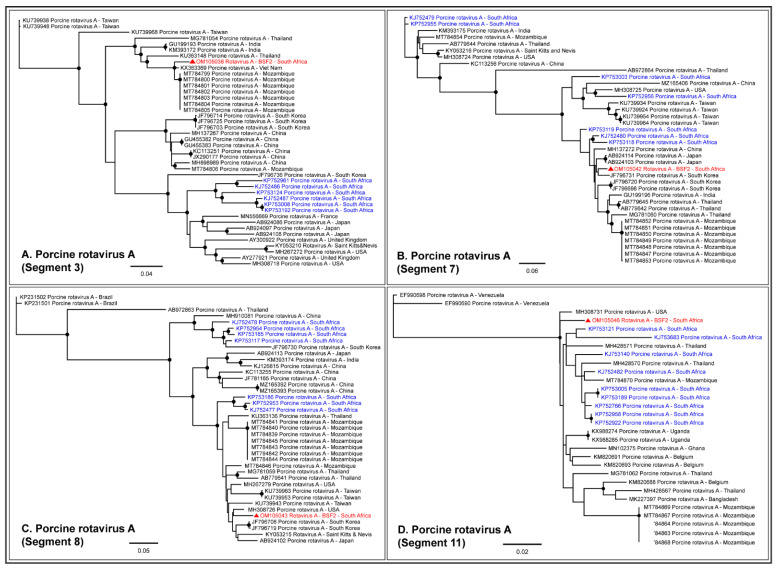

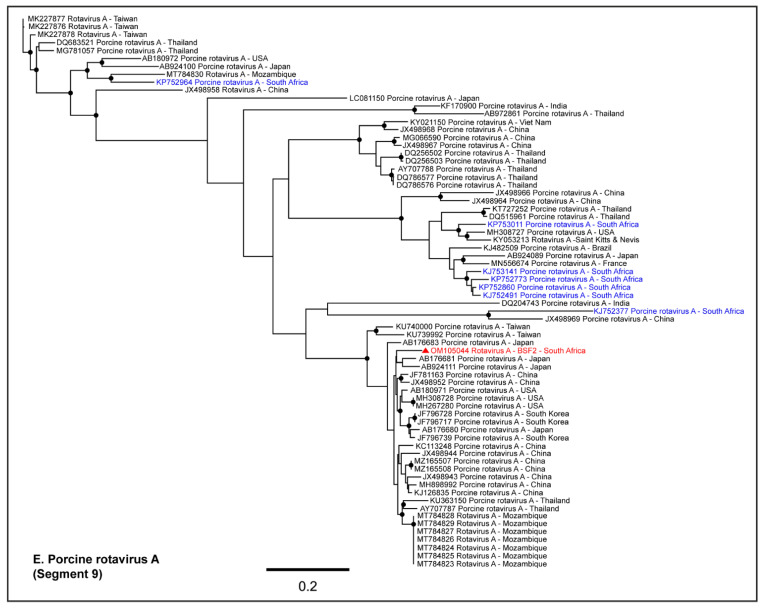
The PhyML phylogenetic analysis of South African RVA sequences. The RVA sequences generated in this study are highlighted in red. The other available RVA sequences from South African swine at NCBI-GenBank are highlighted in blue. (**A**–**E**) The PhyML tree clustered the South African RVA genomes in separate groups. It is likely that there are multiple RVA genotypes in circulation in the South African swine populations. The black dots at nodes represent ≥80% bootstrap support.

Either partial or complete coding sequences (37.3–98.5% genome coverage) of all the eleven genome segments of RVA were generated from BSF2. In addition, partial sequences of a few segments of RVC (19.8–50.6% genome coverage) and Human Rotavirus B (35.5–75.5% genome coverage) were also generated from BSF2. Briefly, the segment 3 of the RVA genome obtained from the South African BSF2 appeared to be related to the RVA sequences reported from Mozambique (Figure 8A). While segment 7 of the South African swine RVA genome appeared to be related to the genome reported from South Korea (Figure 8B), segment 8 was related to the genome reported from the USA (Figure 8C). Due to the availability of limited sequences for swine RVA genome segment 11, the PhyML tree could not resolve their epidemiology (Figure 8D), however segment 9 clustered with the RVA genomes reported from Japan (Figure 8E). Interestingly, the separate phylogenetic clustering of the RVA genome sequences generated in this study (highlighted in red) and previously reported RVA sequences from the South African swine (highlighted in blue) available at the NCBI-GenBank indicated that there might be multiple RVA genotypes in circulation in the South African swine populations.

**Figure 9:**
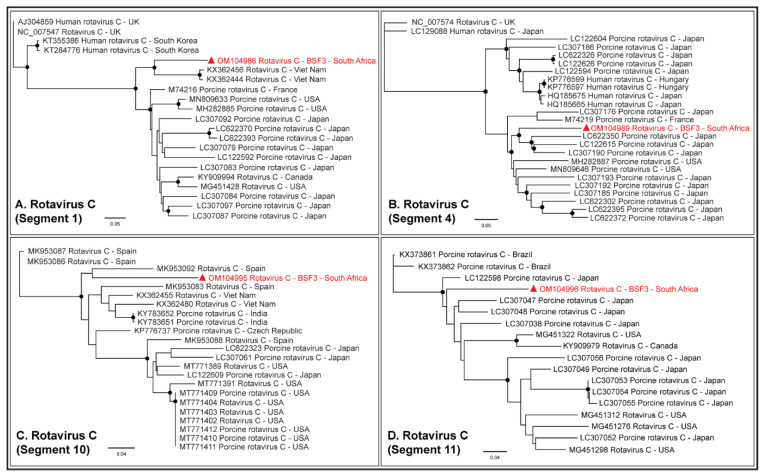
The PhyML phylogenetic analysis of South African RVC genome sequences. (**A**) The South African RVC segment 1 was related to the genome reported from Viet Nam. (**B**) Segment 4 of the South African RVC genome appeared related to the Japanese sequences. (**C**) Segment 10 of the South African RVC genome was related to the genome reported from Spain. (**D**) Segment 11 of the South African RVC genome clustered with the sequences reported from Asia and North America. This might be either due to the circulation of multiple RVC strains in South African backyard swine or reassortments in the RVC genome in the swine. The black dots at nodes represent ≥80% bootstrap support.

While RVA sequences were not obtained at BSF3, partial or complete coding sequences (42.3–99.1% genome coverage) of all the eleven segments of RVC genome were generated from BSF3. The RVC segment 1 generated from South African BSF3 appeared to be closely related to the RVC sequences reported from Viet Nam (Figure 9A). Segment 4 of the South African RVC genome was related to the sequences reported from Japan (Figure 9B). While segment 10 of the South African RVC genome appeared to be related to the genome reported from Spain (Figure 9C), segment 11 clustered with the sequences reported from Asia and North America (Figure 9D). This might be due to the presence of multiple strains of RVC or the reassortment in the RVC genomes in South African backyard swine, which requires further investigation. 

#### 2.1.6. Family Picobirnaviridae

**Figure 10:**
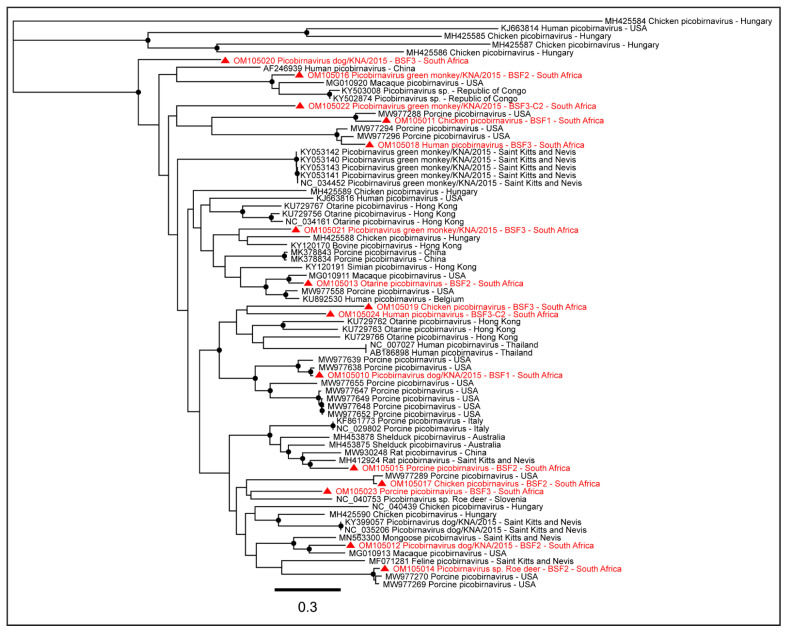
The PhyML phylogenetic analysis of Picobirnavirus sp. The presence of several Picobirnaviruses, including Porcine picobirnavirus, Dog picobirnavirus, Human picobirnavirus, Chicken picobirnavirus, Green monkey picobirnavirus, Otarine picobirnavirus, and Feline picobirnavirus in saliva samples of South African backyard swine suggested active circulation of these viruses at the backyard swine farms. The black dots at nodes represent ≥80% bootstrap support.

Either partial or complete coding sequences of Dog picobirnavirus segment 2 (57.5–97.2% genome coverage) and Chicken picobirnavirus segment 2 (32.8–85.4% genome coverage) were generated from all the three BSFs. The complete coding sequence of Otarine picobirnavirus segment 2 (97.7%) was obtained from BSF2. Partial or near-complete coding sequences of Roe deer picobirnavirus segment 2 (28.7–97.1% genome coverage) were obtained from BSF2 and BSF3. Partial or complete coding sequences of Porcine picobirnavirus segment S (38.3–96.8% genome coverage), partial coding sequences of Green monkey picobirnavirus (32.2–84.9% genome coverage), and Human picobirnavirus segment 2 (32.7–79.9% genome coverage) were obtained from BSF2 and BSF3 (Figure 10). The presence of multiple Picobirnavirus species, especially Dog picobirnavirus, Chicken picobirnavirus, Green monkey picobirnavirus, Roe deer picobirnavirus, and Feline (Cat) picobirnavirus in saliva samples of South African backyard swine suggested the possible interactions of the backyard swine with other wild and domestic animal species.

#### 2.1.7. Family Caliciviridae

**Figure 11:**
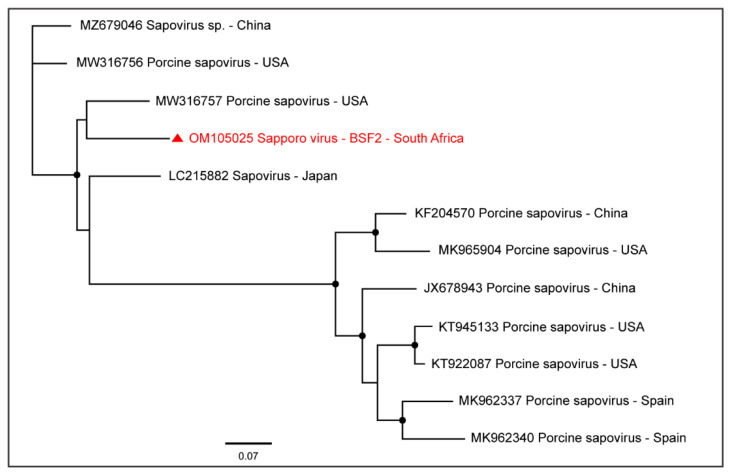
The PhyML phylogenetic analysis determined that the South African genome of Sapporo virus was related to the sequences reported from the USA. The black dots at nodes represent ≥80% bootstrap support.

A near-complete coding sequence of Sapporo virus (99.5% genome coverage; 7290 nt) was obtained from BSF2 which appeared to be related to the Sapporo virus genome reported from the USA (Figure 11). Sapporo virus sequences were not obtained from the other two BSFs. 

#### 2.1.8. Family Virgaviridae

**Figure 12:**
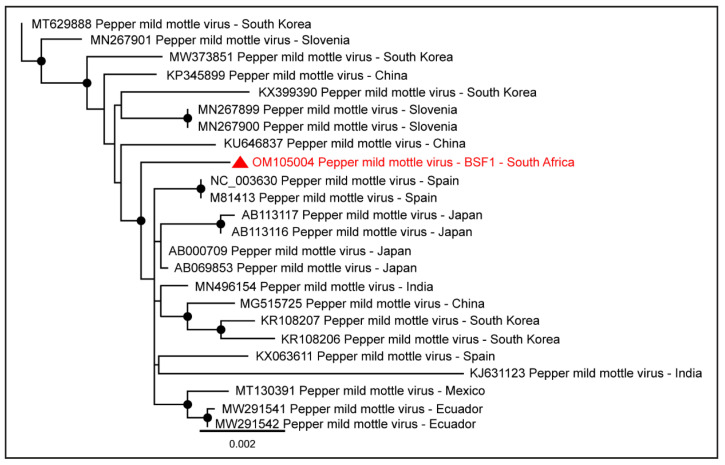
The PhyML phylogenetic tree of Pepper mild mottle virus obtained from the saliva sample of the backyard swine at South African BSF1. The black dots at nodes represent ≥80% bootstrap support.

Genome sequences of plant viruses were also obtained from BSF1 and BSF2. Near-complete coding sequences of Pepper mild mottle virus (98.0% genome coverage; 6233 nt and 88.6% genome coverage; 5632 nt) were obtained from BSF1 and BSF2, respectively. The Pepper mild mottle virus genome generated from the saliva of the backyard swine at BSF1 clustered with the sequences reported from Asia, Europe, and Americas (Figure 12). 

Due to the absence of the full-length Pepper mild mottle virus genome sequences from South Africa and the availability of the limited sequences from other geographical locations, the epidemiology of Pepper mild mottle virus genome generated from the backyard swine at BSF1 could not be determined. In addition, partial coding sequences of the Tobacco mild green mosaic virus (80.4% genome coverage; 5108 nt and 17.1% genome coverage; 1084 nt) were generated from BSF1 and BSF2, respectively. 

#### 2.1.9. Family Phenuiviridae

A partial coding sequence of Dipteran hudivirus segment 3 (47.9% genome coverage; 592 nt) was generated from BSF1. A partial coding sequence of Rice stripe tenuivirus (32.9% genome coverage; 709 nt) was obtained from BSF3. 

#### 2.1.10. Family Dicistroviridae

The partial coding sequence of Goose dicistrovirus (39.8% genome coverage; 3636 nt) and Aphid lethal paralysis virus (20.5% genome coverage; 2007 nt) were obtained from BSF2. 

#### 2.1.11. Family Partitiviridae

The partial coding sequences of Aspergillus fumigatus partitivirus 2 (55.6% genome coverage; 1013 nt) and Penicillium stoloniferum virus S (20.3% genome coverage; 356 nt) were generated from BSF2. 

#### 2.1.12. Family Tobaniviridae

A partial coding sequence of Porcine torovirus (33.7% genome coverage; 9528 nt) was obtained from BSF3. 

#### 2.1.13. Family Retroviridae

A partial coding sequence of Koala retrovirus (22.2% genome coverage; 1869 nt) was obtained from BSF3. 

#### 2.1.14. Family Tombusviridae

The partial coding sequence of Johnsongrass chlorotic stripe mosaic virus (93.0% genome coverage; 4112 nt) was obtained from BSF1. 

#### 2.1.15. Unclassified RNA Viruses

The partial coding sequences of five unclassified RNA viruses, including Beihai tombus-like virus 15 (40.9% genome coverage; 1361 nt), Thika virus (39.6% genome coverage; 3603 nt), and Hubei picorna-like virus 15 (39.6% genome coverage; 3926 nt) were generated from BSF2. Partial coding sequences of Hubei tombus-like virus 17 (49.9% genome coverage; 1854 nt) and Wuhan insect virus 23 (26.2% genome coverage; 387 nt) were obtained from BSF3. 

Overall, the present study identified a high diversity of swine oral RNA virome in backyard swine saliva samples under investigation. A summary of the genome characterizations is provided in Supplementary Table S1. 

### 2.2. NCBI-GenBank Accession Numbers of the Virus Genomes Generated in the Present Study

The virus genome sequences generated in this study can be accessed at NCBI-GenBank with the accession numbers provided in Table 2. 

## 3. Discussion

In a previous molecular study aimed at detecting the prevalence of IAV in backyard swine populations in the uMgungundlovu District of the KwaZulu-Natal province of South Africa, using real-time RT-PCR, we did not find IAV RNA in samples under investigation. However, some of the swine did show disease symptoms such as coughing, sneezing, lethargy, and red patches on the skin, which warranted further analysis of a subset of the swine saliva samples to explore their oral virome. Metagenomic analysis of the RNA fraction was performed on one saliva sample from each of the three backyard farms under investigation. While the healthy swine saliva taken from BSF1 had only a few RNA viruses, the other two pooled saliva samples taken from BSF2 and BSF3 from swine having clinical signs of disease generated a wide range of RNA viruses (Figure 1). Interestingly, viruses of the family *Astroviridae* were prevalent, according to the number of reads; genomes of PAstV-2, PAstV-4, Astrovirus wild boar, Porcine bastrovirus, Mamastrovirus 2, and Mamastrovirus 3 were identified. Most of these enteroviruses are endemic to swine populations worldwide and are associated with diarrhoea in swine, including neonatal piglets [43,44]; however, they have also been detected in healthy swine in all age groups [45,46]. In particular, PAstV-2 and PAstV-4 have been frequently reported in swine populations in Asia [47,48]. These viruses have a single-stranded, positive-sense RNA genome comprising three open reading frames (ORFs); ORF1ab, ORF1a, and ORF2 [49]. While ORF1ab encodes the nonstructural protein, the ORF1a encodes RNA-dependent RNA polymerase (RdRp) and the ORF2 encodes the capsid protein, which is highly divergent due to the immune pressure from the host [49]. 

Intriguingly, in a recent study PAstV-4 was found to be associated with acute respiratory illness in piglets in Oklahoma, USA [50]. This might explain our observation of the piglets at the BSF2 with clinical signs of coughing and sneezing that were found to be infected with PAstV-4 in this study; however, this requires further investigation for confirmation. Interestingly, PAstV-4 sequences were also detected in grower swine at the BSF3, which did not exhibit any clinical signs of respiratory illness; instead, these swine had red patches on the skin, which may be associated with other sources of infection and therefore requires further investigation. Here it should be noted that the piglets infected with PAstV-4 that had coughing and sneezing illness at BSF2 were about three months old, while the grower pigs at BSF3 were about five to six months old. While the present study could not determine whether PAstV-4 infection manifests as clinical respiratory signs in piglets and not in the older swine, it remains a question for further investigation to ascertain whether these viruses are incidental findings or are indeed associated with the clinical signs. It was noteworthy that while swine saliva samples collected from BSF2 and BSF3 had several virus species that were common in these swine, they exhibited different disease symptoms. Therefore, it appears that the clinical signs of illness in swine at BSF2 and BSF3 might not be related to the prevalence of endemic swine viruses; instead, they would be the result of environmental factors, including improper hygiene or feed and water, among others. Here it is important to note that several of these enteric viruses, such as astroviruses and picornaviruses, are endemic to swine populations globally [46]; therefore, their relatedness with previously reported genomes from different geographical locations might be due to swine trade or breeding.

Genome sequences of several members of the family *Picornaviridae* were obtained in this study, including Porcine kobuvirus, Porcine teschovirus A, Porcine sapelovirus 1, Porcine enterovirus 9, Pasivirus A, and Tottorivirus A. These viruses have a monopartite, positive-sense, single-stranded RNA genome and have been associated with swine gastrointestinal, reproductive, respiratory, and neurologic diseases; however, the infections have also been reported to be subclinical [51]. Porcine enteric sapovirus was also detected in this study, which has previously been detected in clinically healthy as well as diarrhoeic pigs in various age groups [52], including 15-day-old neonatal piglets [53]. 

Genome sequences of several viruses from the Order Picornavirales were obtained, including Posavirus 1, Posavirus 3, Picornavirales Bu-1, and Picornavirales Tottori-HG1. These are porcine stool-associated RNA viruses with a positive-sense single-stranded RNA genome [54]. They have been reported in swine populations in various age groups causing diarrhoea; however, infections may also be subclinical [55,56,57]. 

Several species of Picobirnaviruses that were identified from either partial or complete coding sequences of the RdRp gene (RNA segment 2) of Picobirnaviruses included Porcine picobirnavirus, Dog picobirnavirus, Human picobirnavirus, Chicken picobirnavirus, Green monkey picobirnavirus, Otarine picobirnavirus, Roe deer picobirnavirus, and Feline (Cat) picobirnavirus. These are enteric viruses and have been detected in the feces of various mammalian species and humans with or without diarrhoeic diseases [58,59]. These are primarily opportunistic enteric pathogens found in birds and mammals [59,60,61]; the avian host may serve as the carrier of these Picobirnaviruses [62,63]. Picobirnaviruses have a bi-segmented double-stranded RNA genome [60] in which RNA segment 1 encodes the capsid protein, and segment 2 encodes the RdRp gene [58]. The presence of different species of Picobirnaviruses in backyard swine suggested a widespread circulation of these viruses in animals and birds, possibly transmitted to swine through direct contact on the backyard farms. A high prevalence and diversity of Picobirnaviruses were also recently reported in swine populations on Saint Kitts and Nevis Island in the Caribbean [58] and Hong Kong [64]. Our observation of Otarine picobirnavirus in backyard swine was intriguing, since the primary host of the Otarine picobirnavirus is an aquatic mammal, the California sea lion, from which the first genome sequence of Otarine picobirnavirus was generated in Hong Kong [65]. Due to limited information on the Otarine picobirnavirus, the transmission dynamics remain obscure, and therefore, the possible route of transmission of Otarine picobirnavirus to the backyard swine in the KwaZulu-Natal province of South Africa could not be ascertained. Interestingly, in a recent study, we reported on the possible dissemination of avian IAV subtypes to swine populations worldwide, [66] suggesting the potential role of migratory birds in the dissemination of these RNA viruses [67,68]. It should be noted that the present study was conducted in a coastal area of South Africa where aquatic birds are frequently seen; therefore, if the occurrence of Otarine picobirnavirus in the backyard swine was related to the migration of aquatic avian species, this remains a question for further investigation. 

Taken together, the presence of swine enteric viruses, such as astroviruses, picornaviruses, picobirnaviruses, RVA, RVC, and Human rotavirus B in the backyard swine saliva suggest that the backyard swine are likely at risk of developing diarrhoeic diseases [43,47]. In addition, the possibility of zoonotic transmission of RVA and RVC from swine to the exposed household members or workers also poses a risk of diarrhoeic disease in these contacts because the rotaviruses, such as RVA and RVC, have been reported to have a zoonotic propensity. For example, Ianiro et al., 2019 reported zoonotic transmission of RVA from swine to a child in Italy who was hospitalized due to gastroenteritis [32] while another long-term study identified eight RVA strains that were zoonotically transmitted to hospitalized pediatric patients over a 15-year period in Hungary [69]. In addition, Tacharoenmuang et al., 2021 reported swine-to-human transmission of RVA in Thailand, which caused severe diarrhoea in the infected human population [70] while the zoonotic transmission of RVC was reported from swine to children causing diarrhoea in Brazil [71]. While RVA, RVB, and RVC infections have been previously reported in diarrhoeic swine populations in South Africa [37,38], only RVA genome sequences were available in the NCBI-GenBank and therefore were included in our analysis. Various South African swine RVA genomes appeared to be distantly related to each other, including the RVA genomes generated in the present study. These observations indicated that there might be multiple RVA genotypes circulating in swine populations in South Africa. Since rotavirus infection in swine might also remain subclinical, as we observed in the present investigation, their actual disease burden in South African swine populations might be underestimated.

Our finding of HEV in the South African backyard swine was not surprising because HEV has been reported in swine populations in various age groups in several countries [72,73], including South Africa [42]. An experimental study determined that the fecal shedding of HEV in swine starts 12.6 days post-infection and may last up to 10.5 days in contact-infected swine [72]; however, the length of HEV fecal shedding was reported to be dependent upon the route as well as the dose of infection, and the coinfection status of the swine with other viral pathogens. When HEV infection was administered orally or through contact infection, the length of HEV fecal shedding in swine was 9.7 days [72,74]. The fecal shedding of HEV on backyard farms may predispose other species, including humans, to HEV infection. In addition, the environmental factors that might enhance HEV persistence within the backyard farm include fomites, contaminated water, and feed [75]. Since the zoonotic transmission of HEV from swine to humans is possible either through contact or the consumption of undercooked pork meat, [72] adequate precautions should be taken to avoid this. Since HEV causes subclinical infection in swine [75], it might facilitate the zoonotic spillover of HEV to the exposed human populations and therefore poses a serious threat to public health.

The presence of plant viruses, including Pepper mild mottle virus, Tobacco mild green mosaic virus, and Johnsongrass chlorotic stripe mosaic virus in backyard swine saliva samples, suggested a possibility of feed-related transmission of these viruses. A plant virus genome was also detected in a recent backyard swine fecal virome study in Mexican swine [76]. The partial sequences of a few other viruses, including fungal and unclassified RNA viruses, suggested a limited transmission of these viruses on the backyard swine farms.

Overall, this study reported a high diversity of swine enteric viruses on the backyard swine farms under investigation. While most of these enteric viruses are endemic in swine populations globally, some of the picobirnaviruses appeared to have been transmitted from other animals present on the farm, based on the phylogenetic analysis. The persistent direct interaction between backyard swine and the household members without the necessary biosecurity measures in place would enhance the disease burden in backyard swine populations and simultaneously threaten the health and well-being of the household members. Information on the RNA virome of backyard swine is imperative to assess the risk of disease outbreaks in swine farming and rural communities. While RVA, RVB, RVC, and HEV have previously been reported from commercial swine farms in South Africa, this is the first study to report the oral RNA virome of backyard swine in the country. The present study would serve as a reference for future investigations of virus pathogens in swine populations in South Africa.

## 4. Materials and Methods

### 4.1. Ethics Approval 

We obtained the full approval of the research protocol from the Animal Research Ethics Committee of the University of KwaZulu-Natal; Reference# AREC/041/019D. We obtained the Section 20 permit from the Department of Agriculture, Land Reform and Rural Development (DALRRD), South Africa, in terms of the Animal Diseases Act, 1984 (Act No. 35 of 1984); Reference# 12/11/1/5/4 (1425 AC) (1). 

### 4.2. Sample Collection and Processing 

We used three backyard swine oral secretion (saliva) samples initially collected for IAV detection in March 2021 from three distantly located backyard swine farms in the uMgungundlovu District of the KwaZulu-Natal province, South Africa. The BSFs under investigation were identified in consultation with the State Veterinary Department of Pietermaritzburg, KwaZulu-Natal. The criteria of the BSF selection were based on the backyard farm size (number of sows), years in operation, and distance from each other to represent different areas of the uMgungundlovu District. We conducted a passive surveillance, and a written informed consent was obtained from each backyard farmer who agreed for a voluntary participation in the study. The backyard swine under investigation were visually screened with the assistance of an Animal Health Technician (AHT) from the State Veterinary Department. Our strategy was to obtain the saliva samples primarily from swine with clinical signs of influenza-like illness or any other apparent disease symptoms. In cases where the swine under investigation did not exhibit any signs of illness and therefore appeared healthy, we also sampled them. 

The first backyard swine farm (BSF1) had only been established a year before the commencement of this study and had apparently healthy sows and piglets with no clinical signs of disease. We chose a saliva sample that was obtained from a one-year-old apparently healthy swine at BSF1 because the piglets at this farm were quite young and therefore did not yield enough sample volume. The second backyard swine farm (BSF2) was more than four years old and had apparently healthy sows, although some piglets exhibited influenza-like illness, including coughing and sneezing. We chose one pooled saliva sample obtained from BSF2, taken from multiple three-month-old piglets with clinical signs of coughing and sneezing. While the rope was chewed by at least seven piglets that were enclosed within the pen, most of them had apparent clinical signs of illness. The third backyard swine farm (BSF3) was also more than four years old and had apparently healthy sows and piglets, although some growers had red patches on their skin. We chose a pooled saliva sample taken from five-month-old growers with red patches on the skin. While seven growers chewed the rope, most of them had red patches on their skin.

We collected the saliva of backyard swine using a standard non-invasive hanging rope method [77,78]. Briefly, about 80 cm long three-strand twisted 100% cotton rope was suspended in the air inside the pens and the swine were allowed to chew the rope for about 15 min, after which the rope was taken away and squeezed aseptically into a plastic Ziploc bag and the saliva was transferred to a 15 mL Falcon tube. The saliva samples were transported on dry ice to a BSL2 laboratory at the University of KwaZulu-Natal, Durban, where they were centrifuged at 1500× *g* for 10 min at 4 °C to eliminate feed contaminants. The saliva was then aliquoted into 2 mL cryovials and stored in a −80 °C freezer until processed.

For deep sequencing, where available, we chose pooled saliva samples obtained from the piglets and growers confined in multiple numbers within pens with clinical signs of illness to better assess the diversity of their oral RNA virome. However, the saliva sample chosen from the BSF1 belonged to one of the apparently healthy adult swine kept in separate pens, as there was insufficient volume of saliva available from the pooled piglet samples. 

### 4.3. Metagenomic Sequencing

For metagenomic analysis, the saliva samples were shipped on dry ice to the Biotechnology Platform Laboratory of Agricultural Research Council (ARC), Onderstepoort, Pretoria, South Africa, for deep sequencing. Briefly, the viral RNA was extracted using the NucleoMag Pathogen kit (Macherey-Nagel, Dueren, Germany). The library preparation was performed using the Illumina Stranded Total RNA Prep according to the manufacturer’s specifications. Deep sequencing was performed on an Illumina HiSeq X instrument (San Diego, CA, USA). 

### 4.4. Genome Assembly 

The FASTQ files containing paired-end reads were analyzed using Genome Detective v 1.135 (www.genomedetective.com; accessed on 7 September 2021) [79]. Briefly, Genome Detective performed quality control, trimmed the raw sequence reads, filtered out non-viral reads, and sorted the remaining reads into groups. Each group was assigned a unique taxonomic ID representing the lowest common ancestor (LCA). The reads in each group were de novo assembled, and the resulting contigs were verified using reference genomes available in the NCBI database to determine percent genome coverage and percent nucleotide and amino acid identities. The Genome Detective aligned the contigs to the reference genomes using the codon-aware pairwise alignment tool, Annotated Genome Aligner (AGA) [80], to calculate the consensus sequence. We then manually performed nucleotide BLAST analysis to verify the virus contigs obtained in the study. Later, we manually analyzed all the virus contigs in ‘Geneious Prime 2021.2.2’ software (Auckland, New Zealand) to determine insertions and deletions compared to the reference genome. 

### 4.5. Phylogenetic Analysis

Nucleotide sequences were aligned using the MUSCLE algorithm in MEGA-X software [81] to find the best model for the phylogenetic analysis. The GTR + G + I model was used to construct Maximum Likelihood (ML) trees using PhyML [82] in ‘Geneious Prime 2021.2.2’. A bootstrap analysis was performed using 1000 bootstrap replications. Phylogenetic analyses were conducted for those virus genomes which generated complete or near complete coding sequences in the present study.

## 5. Conclusions

A high prevalence of PAstV-4, PAstV-2, and other swine enteric viruses, including rotaviruses, pose a significant risk of diarrhea and other associated diseases in the backyard swine populations. In addition, the presence of HEV in backyard swine saliva poses the risk of zoonotic transmission to the exposed humans. Our evidence suggests that HEV and rotaviruses may be currently circulating in backyard swine populations, remaining undetected due to subclinical infections in swine. Therefore, maintaining hygiene and avoiding direct contact with swine feces and saliva is recommended to avoid the zoonotic transmission of these viruses. The countrywide molecular surveillance in backyard swine populations should be implemented to explore the diversity of RNA viruses in other backyard swine holdings in South Africa.

## Figures and Tables

**Table 1:** Near full-length genomes generated from backyard swine saliva samples in the present study.

Virus ID	Percent Coverage of Virus Genomes		
	BSF1	BSF2	BSF3
Hepatitis E virus	-	-	97.7%
Mamastrovirus 3	-	97.9%	-
Pasivirus A	-	99.3%	96.3%
Pepper mild mottle virus	98.0%	-	-
Picornavirales Tottori-HG1	-	-	99.2%
Porcine astrovirus 2	99.3%	99.3%	96.3%
Porcine bastrovirus	-	-	97.3%
Porcine enterovirus 9	-	99.3%	-
Porcine enteric sapovirus	-	99.5%	-
Porcine kobuvirus	-	98.9%	-
Posavirus 1	-	99.2%	99.2%
Posavirus 3	-	-	99.5%
Sapelovirus A	-	98.9%	98.9%
Teschovirus A	-	98.3%	99.4%

BSF = Backyard swine farm.

**Table 2:** The NCBI-GenBank accession numbers of the genome sequences generated from the backyard swine saliva samples in the present study.

Name of Virus	Backyard Farm ID	Genome Length (# Nucleotides) *	GenBank Accession Numbers (This Study)
Porcine astrovirus 4	BSF3	4492	OM104029
Astrovirus wild boar/WBAstV-1/2011/HUN	BSF3	4854	OM104030
Mamastrovirus 2	BSF3	5338	OM104031
Dromedary astrovirus	BSF3	2542	OM104032
Porcine bastrovirus	BSF3	5848	OM104033
Hepatitis E virus	BSF3	7040	OM104034
Picornavirales Tottori-HG1	BSF3	9787	OM104035
Pasivirus A	BSF3	6672	OM104036
Posavirus 1	BSF3	9782	OM104037
Posavirus 3	BSF3	8845	OM104038
Porcine sapelovirus 1	BSF3	7438	OM104039
Picornavirales Bu-1	BSF3	4499	OM104040
Rotavirus C—segment 1	BSF3	3279	OM104986
Rotavirus C—segment 2	BSF3	1305	OM104987
Rotavirus C—segment 3	BSF3	1001	OM104988
Rotavirus C—segment 4	BSF3	2105	OM104989
Rotavirus C—segment 5	BSF3	653	OM104990
Rotavirus C—segment 6	BSF3	937	OM104991
Rotavirus C—segment 7	BSF3	1023	OM104992
Rotavirus C—segment 8	BSF3	365	OM104993
Rotavirus C—segment 9	BSF3	439	OM104994
Rotavirus C—segment 10	BSF3	615	OM104995
Rotavirus C—segment 11	BSF3	432	OM104996
Rotavirus C—segment 1	BSF2	835	OM104997
Rotavirus C—segment 4	BSF2	845	OM104998
Rotavirus C—segment 6	BSF2	376	OM104999
Rotavirus C—segment 7	BSF2	302	OM105000
Rotavirus C—segment 8	BSF2	294	OM105001
Porcine kobuvirus	BSF2	8123	OM105002
Porcine astrovirus 2 (contig 2)	BSF1	3874	OM105003
Pepper mild mottle virus	BSF1	6233	OM105004
Johnsongrass chlorotic stripe mosaic virus	BSF1	1643	OM105005
Posavirus 1	BSF1	4873	OM105006
Tobacco mild green mosaic virus	BSF1	1220	OM105007
Aspergillus fumigatus partitivirus 2	BSF2	1391	OM105008
Penicillium stoloniferum virus S	BSF2	356	OM105009
Picobirnavirus dog/KNA/2015—segment 2	BSF1	1594	OM105010
Chicken picobirnavirus—segment 2	BSF1	1496	OM105011
Picobirnavirus dog/KNA/2015—segment 2	BSF2	1651	OM105012
Otarine picobirnavirus—segment 2	BSF2	1655	OM105013
Picobirnavirus sp. Roe deer—segment 2	BSF2	1644	OM105014
Porcine picobirnavirus—segment S	BSF2	1669	OM105015
Picobirnavirus green monkey/KNA/2015—segment 2	BSF2	679	OM105016
Chicken picobirnavirus—segment 2	BSF2	707	OM105017
Human picobirnavirus—segment 2	BSF3	1428	OM105018
Chicken picobirnavirus—segment 2	BSF3	1276	OM105019
Picobirnavirus dog/KNA/2015—segment 2	BSF3	597	OM105020
Picobirnavirus green monkey/KNA/2015—segment 2	BSF3	467	OM105021
Picobirnavirus green monkey/KNA/2015—segment 2 (contig 2)	BSF3	611	OM105022
Porcine picobirnavirus—segment S	BSF3	748	OM105023
Human picobirnavirus—segment 2 (contig 2)	BSF3	574	OM105024
Sapporo virus	BSF2	7290	OM105025
Tottorivirus A	BSF2	6746	OM105026
Pasivirus A	BSF2	6876	OM105027
Porcine enterovirus 9	BSF2	7351	OM105028
Teschovirus A	BSF2	7001	OM105029
Porcine sapelovirus 1	BSF2	7473	OM105030
Picornavirales Bu-1	BSF2	8877	OM105031
Porcine astrovirus 4	BSF2	4508	OM105032
Astrovirus wild boar/WBAstV-1/2011/HUN	BSF2	4451	OM105033
Dromedary astrovirus	BSF2	2580	OM105034
Mamastrovirus 3	BSF2	6562	OM105035
Rotavirus A—segment 1	BSF2	1388	OM105036
Rotavirus A—segment 2	BSF2	1054	OM105037
Rotavirus A—segment 3	BSF2	2552	OM105038
Rotavirus A—segment 4	BSF2	734	OM105039
Rotavirus A—segment 5	BSF2	599	OM105040
Rotavirus A—segment 6	BSF2	689	OM105041
Rotavirus A—segment 7	BSF2	999	OM105042
Rotavirus A—segment 8	BSF2	990	OM105043
Rotavirus A—segment 9	BSF2	1024	OM105044
Rotavirus A—segment 10	BSF2	485	OM105045
Rotavirus A—segment 11	BSF2	585	OM105046
Mamastrovirus 2	BSF1	3401	OM105047
Porcine astrovirus 2	BSF1	6364	OM105048
Posavirus 1	BSF2	9789	OM105049
Teschovirus A	BSF3	7078	OM966657
Porcine astrovirus 2	BSF2	6313	OM966722
Porcine astrovirus 2	BSF3	6127	OM966723

* The largest contig was submitted to the NCBI-GenBank for those genomes where more than one contig was generated for a given genome.

## Data Availability

The virus genome sequences generated in this study are available on NCBI-GenBank and can be accessed with the following accession numbers: OM104029–OM104040, OM104986–OM105049, OM966657, OM966722, and OM966723.

## Supplementary Materials

The following supporting information can be downloaded at: https://www.mdpi.com/article/10.3390/pathogens11080927/s1, Table S1: Overview of virus genome sequences generated from South African backyard swine saliva samples.
