# Supplementary material for: Metagenomic Analysis of RNA Fraction Reveals the Diversity of Swine Oral Virome on South African Backyard Swine Farms in the uMgungundlovu District of KwaZulu-Natal Province
Source: Pathogens. 2022 Aug 17;11(8):927. doi: 10.3390/pathogens11080927 (PMC9416320; doi:10.3390/pathogens11080927)
Supplement: Supplementary file 1 [file pathogens-11-00927-s001.zip › pathogens-1814655-supplementary.pdf]

Supplementary Table S1: Overview of virus genome sequences generated from South African backyard swine saliva samples

Virus ID (Backyard Farm ID)	Genome characterization						
	**#Contigs**	**Percent coverage**	**Reference genome**	**Percent identity**	**#Nucleotides (annotation of genome segments)**	**Insertion (amino acid position)**	**Deletion (amino acid position)**
**Family *Astroviridae***							
Porcine astrovirus type-2- contig 1 (BSF1)	1	99.3	NC023674	nt (70.1)<br>aa (75.6)	6364 nt (complete coding regions for ORF1ab, ORF1a, ORF2)	**ORF1a:** K (396)<br>**ORF2:** Q (8), S (286), I (315), N (366), S (401), Y (422), HG (482), GNGY (539), DVVT (626), E (639), PPPSEE (653), EASNFYDFDSDL (665)	**ORF1a:** I (626)<br>**ORF2:** S (36), R (42), A (43), G (435), L (489)
Porcine astrovirus type-2- contig 2 (BSF1)	1	65.4	NC023674	nt (80.8)<br>aa (88.9)	4130 nt (complete coding regions for ORF 1ab and ORF 1a, partial coding region for ORF2)	None	**ORF1ab:** L (1281), A (1282)
Porcine astrovirus type-2 (BSF2)	7	82.1	NC023674	nt (79.1)<br>aa (85.9)	5185 nt (complete coding region for ORF1ab, partial coding regions for ORF1a, ORF2)	**ORF2:** V (535), ECS (540), VS (629), PPPED (653), 665 (E),	None
Porcine astrovirus type-4 (BSF2)	4	96.2	NC023675	nt (80.9)<br>aa (84.0)	6384 nt (complete coding regions for ORF1ab and ORF1a, partial coding region for ORF2)	None	**ORF1a:** T (603)
Astrovirus wild boar (BSF2)	7	92.7	NC016896	nt (81.5)<br>aa (81.5)	6219 nt (complete coding regions for ORF1ab and ORF1a, partial coding region for ORF2)	**ORF1ab:** LV (226)<br>**ORF2:** E (756)	**ORF1ab:** H (214), R (233), R (234) G (235), G (236)<br>**ORF1a:** T (398)<br>**ORF2:** G (718), A (721)
Dromedary astrovirus (BSF2)	2	89.7	NC027711	nt (74.0)<br>aa (80.4)	5674 nt (complete coding regions for ORF1a and ORFX, partial coding regions for ORF1ab and ORF2)	**ORF2:** P (9), T (401), Q (491), M (505), HNNN (542), E (554), GT (625), INA (638), PEEET (671)	**ORF2:** T (426)
Porcine astrovirus type-4 (BSF3)	4	77.0	NC023675	nt (85.2)<br>aa (87.3)	5111 nt (complete coding regions for ORF1ab and ORF1a, partial coding region for ORF2)	None	None
Porcine astrovirus type-2 (BSF3)	8	70.0	NC023674	nt (80.7)<br>aa (85.5)	4421 nt (complete coding region for ORF1ab, partial coding regions for ORF1a, ORF2)	ORF2: NNYS (540)	None
Astrovirus wild boar (BSF3)	4	87.0	NC016896	nt (83.5)<br>aa (84.5)	5833 nt (complete coding regions for ORF1ab and ORF1a, partial coding region for ORF2)	**ORF1ab:** LM (226),	**ORF1ab:** R (233), R (234) G (235), G (236)<br>**ORF1a:** T (398)

							**ORF2:** L (708)
Dromedary astrovirus (BSF3)	2	47.1	NC027711	nt (79.4) aa (85.7)	2979 nt (complete coding region for ORF1a, partial coding region for ORF1ab)	None	None
Bovine astrovirus (BSF2)	1	29.3	NC024297	nt (62.2) aa (58.8)	1829 nt (partial coding sequence for capsid protein precursor)	**Capsid protein:** S (304), T (422), ADTNH (437), RADG (655), SFGC (665), L (679)	**Capsid protein:** P (387)
Mamastrovirus 2 (BSF1)	1	54.1	NC034974	nt (75.1) aa (74.5)	3401 nt (complete coding sequence for capsid protein, partial coding sequence for RdRp)	**Capsid protein:** N (365), K (454), S (515), TNSS (547), NT (601)	**Capsid protein:** S (10), R (42), T (43), P (319), A (342), V (374), Q (375), T (408), P (409), P (410), G (411), N (449), G (458), L (564), Y (565), G (619), L (620), T (642), T (643), L (644), R (645), V (646), G (647)
Mamastrovirus 2 (BSF2)	4	77.5	NC034974	nt (80.4) aa (84.1)	4920 nt (complete coding sequence for RdRp, partial coding sequence for capsid and nonstructural proteins)	None	**Capsid protein:** T (43)
Mamastrovirus 3 (BSF2)	1	97.9	NC025379	nt (84.6) aa (91.4)	6562 nt (complete coding sequence)	**Capsid protein:** D (643)	**Capsid protein:** A (662), S (663)
Porcine bastrovirus (BSF3)	1	97.4	NC032423	nt (81.6) aa (91.8)	5848 nt (complete coding sequence)	**Structural polyprotein:** KQWN (464)	**Structural polyprotein:** I (433), P (434), S (643), A (651), G (652), G (653)
**Family *Hepeviridae***							
Hepatitis E virus (BSF3)	1	97.7	NC001434	nt (74.3) aa (84.8)	7040 nt (complete coding sequence for hypothetical protein and capsid protein, partial coding sequence for nonstructural protein)	**Nonstructural protein:** IWV (739), VDVV (748), PSLA (759)	**Nonstructural protein:** A (775) **Hypothetical protein:** R (4)
**Family *Reoviridae***							
Rotavirus A (BSF2)	3	96.1	NC011507	nt (78.9) aa (90.0)	Segment 1 (VP1): 3173 nt- near complete coding sequence	None	None
	2	78.5	NC011506	nt (80.3) aa (91.4)	Segment 2 (VP2): 2114 nt- partial coding sequence	DVTMES (29), KE (340),	None
	1	98.5	NC011508	nt (76.4) aa (83.5)	Segment 3 (VP3): 2552 nt- complete coding sequence	None	None
	3	62.7	NC011510	nt (73.5) aa (79.8)	Segment 4 (Outer capsid spike): 1482 nt- partial coding sequence	None	S (603), S (604), V (605)

	1	37.3	NC011500	nt (64.4) aa (52.0)	Segment 5 (NSP1): 602 nt- partial coding sequence	None	E (152)
	2	75.1	NC011509	nt (79.9) aa (90.9)	Segment 6 (VP6): 1018 nt- partial coding sequence	None	None
	1	95.5	NC011501	nt (75.0) aa (77.9)	Segment 7 (NSP3): 1055 nt- near complete coding sequence	None	G (313), C (314)
	1	93.5	NC011502	nt (79.1) aa (86.5)	Segment 8 (NSP2): 990 nt- complete coding sequence	None	None
	1	96.5	NC011503	nt (78.7) aa (85.9)	Segment 9 (VP7): 1024 nt- complete coding sequence	None	Y (175)
	1	64.6	NC011504	nt (78.4) aa (81.4)	Segment 10 (NSP4): 485 nt- partial coding sequence	None	None
	1	88.2	NC011505	nt (88.9) aa (90.9)	Segment 11 (NSP5 and NSP6): 588 nt- complete coding sequence for NSP6, near-complete coding sequence for NSP5	None	NSP5: N (140)
Rotavirus C (BSF2)	2	38.6	NC007547	nt (85.9) aa (93.4)	Segment 1 (VP1): 1277 nt- partial coding sequence	None	None
	2	19.8	NC007546	nt (85.8) aa (95.9)	Segment 2 (VP2): 542 nt- partial coding sequence	None	None
	2	50.6	NC007574	nt (82.4) aa (85.8)	Segment 4 (VP3): 1095 nt- partial coding sequence	None	None
	2	44.7	NC007543	nt (75.8) aa (72.5)	Segment 6 (NSP3): 603 nt- partial coding sequence	None	None
	1	23.8	NC007544	nt (67.9) aa (61.4)	Segment 7 (NSP1): 302 nt- partial coding sequence	None	None
	1	27.7	NC007571	nt (83.7) aa (83.7)	Segment 8 (VP7): 294 nt- partial coding sequence	None	None
Rotavirus C (BSF3)	1	99.1	NC007547	nt (85.1) aa (90.6)	Segment 1 (VP1): 3279 nt- near complete coding sequence	None	None
	2	79.3	NC007546	nt (82.1) aa (90.9)	Segment 2 (VP2): 2170 nt- partial coding sequence	None	None
	2	82.4	NC007572	nt (71.7) aa (71.1)	Segment 3 (VP4): 1882 nt- partial coding sequence	QY (142)	N (132), N (133), P (209), G (210), I (211), N (212), S (253), K (254), L (255), G (256), D (257), S (390), S (392),

	1	97.1	NC007574	nt (81.8)<br>aa (85.6)	Segment 4 (VP3): 2104 nt- near complete coding sequence	None	None
	2	69.0	NC007570	nt (83.7)<br>aa (90.4)	Segment 5 (VP6): 933 nt- partial coding sequence	None	None
	1	69.4	NC007543	nt (78.5)<br>aa (80.8)	Segment 6 (NSP3): 937 nt- partial coding sequence	None	None
	1	80.6	NC007544	nt (65.5)<br>aa (59.2)	Segment 7 (NSP1): 1023 nt- partial coding sequence	None	None
	2	61.0	NC007571	nt (74.2)<br>aa (76.5)	Segment 8 (VP7): 648 nt- partial coding sequence	SSTL (244)	None
	1	42.3	NC007545	nt (80.4)<br>aa (80.3)	Segment 9 (NSP2): 439 nt- partial coding sequence	None	None
	1	85.1	NC007569	nt (74.1)<br>aa (67.8)	Segment 10 (NS26): 621 nt- partial coding sequence	None	T (131), E (132)
	1	70.5	NC007573	nt (65.7)<br>aa (56.9)	Segment 11 (NSP4): 432 nt- near complete coding sequence	None	None
Human rotavirus B (BSF2)	6	75.5	NC021541	nt (70.7)<br>aa (76.8)	Segment 1 (VP1): 2652 nt- partial coding sequence	None	None
	3	61.5	NC021545	nt (73.1)<br>aa (79.9)	Segment 2 (VP2): 1752 nt- partial coding sequence	R (67)	R (612)
	3	61.3	NC021551	nt (69.6)<br>aa (74.0)	Segment 3 (VP3): 1434 nt- partial coding sequence	None	None
	2	35.5	NC021543	nt (67.2)<br>aa (71.7)	Segment 4 (VP4): 819 nt- partial coding sequence	None	S (315)
	1	59.7	NC021542	nt (61.5)<br>aa (55.3)	Segment 9 (VP7): 486 nt- partial coding sequence	None	H (35)
	1	58.3	NC021550	nt (66.0)<br>aa (69.2)	Segment 10 (NSP4): 438 nt- partial coding sequence	None	None
**Family *Picobirnaviridae***							
Dog picobirnavirus (Segment 2)-Contig 1 (BSF1)	1	94.4	NC035206	nt (62.9)<br>aa (66.3)	Segment 2 (RdRp): 1594 nt- near complete coding sequence	E (178), A (531)	T (514), E (515)
Dog picobirnavirus (Segment 2)-Contig 2 (BSF1)	3	57.5	NC035206	nt (68.8)<br>aa (70.1)	Segment 2 (RdRp): 972 nt- partial coding sequence	L (98)	None

Dog picobirnavirus (Segment 2)-Contig 1 (BSF2)	1	97.2	NC035206	nt (67.1) aa (68.8)	Segment 2 (RdRp): 1642 nt- complete coding sequence	GG (174), G (439)	None
Dog picobirnavirus (Segment 2)-Contig 1 (BSF3)	2	75.7	NC035206	nt (67.8) aa (69.8)	Segment 2 (RdRp): 1278 nt- partial coding sequence	KR (178)	E (168), V (169), V (170), N (171), E (385)
Chicken picobirnavirus (Segment 2)-Contig 1 (BSF1)	1	85.4	NC040439	nt (57.4) aa (57.8)	Segment 2 (RdRp): 1451 nt- partial coding sequence	MEF (169), KAIFL (317), ELDRT (443), NAPR (515)	K (177), Q (511),
Chicken picobirnavirus (Segment 2)-Contig 2 (BSF1)	2	32.8	NC040439	nt (69.6) aa (72.3)	Segment 2 (RdRp): 557 nt- partial coding sequence	K (177), G (181)	None
Chicken picobirnavirus (Segment 2)-Contig 1 (BSF2)	3	69.1	NC040439	nt (63.6) aa (69.1)	Segment 2 (RdRp): 1175 nt- partial coding sequence	PTAS (162), DV (179)	None
Chicken picobirnavirus (Segment 2)-Contig 1 (BSF3)	1	73.5	NC040439	nt (60.8) aa (63.8)	Segment 2 (RdRp): 1249 nt- partial coding sequence	NNGSSEAARI (171)	Y (165)
Chicken picobirnavirus (Segment 2)-Contig 2 (BSF3)	1	63.6	NC040439	nt (66.9) aa (71.3)	Segment 2 (RdRp): 1082 nt- partial coding sequence	HA (177), G (440)	K (289), D (290)
Otarine picobirnavirus (Segment 2)-(BSF2)	1	97.7	NC034161	nt (68.7) aa (70.0)	Segment 2 (RdRp): 1650 nt- complete coding sequence	H (41)	None
Roe deer picobirnavirus (Segment 2)-Contig 1- (BSF2)	1	97.1	NC040753	nt (64.3) aa (64.2)	Segment 2 (RdRp): 1671 nt- near complete coding sequence	T (172)	V (10), I (11), A (19), R (534)
Roe deer picobirnavirus	2	43.8	NC040753	nt (61.7) aa (61.4)	Segment 2 (RdRp): 753 partial coding sequence	None	V (10), I (11), K (21), D (335)

(Segment 2)- Contig 2- (BSF2)							
Roe deer picobirnavirus (Segment 2)- Contig 3- (BSF2)	2	28.7	NC040753	nt (66.6) aa (70.1)	Segment 2 (RdRp): 494 partial coding sequence	None	None
Roe deer picobirnavirus (Segment 2)- Contig 1 (BSF3)	2	39.0	NC040753	nt (63.7) aa (59.1)	Segment 2 (RdRp): 671 partial coding sequence	YYNYWTLD (18)	None
Roe deer picobirnavirus (Segment 2)- Contig 2- (BSF3)	3	46.8	NC040753	nt (65.5) aa (67.9)	Segment 2 (RdRp): 805 partial coding sequence	None	None
Porcine picobirnavirus (Segment S)- Contig 1 (BSF2)	1	96.8	NC029802	nt (72.1) aa (73.7)	Segment S (RdRp): 1675 nt- complete coding sequence	Q (536)	R (9), S (10), R (11), D (12),
Porcine picobirnavirus (Segment S)- Contig 2 (BSF2)	2	61.1	NC029802	nt (66.1) aa (72.9)	Segment S (RdRp): 1057 nt- partial coding sequence	GNGAS (167)	None
Porcine picobirnavirus (Segment S)- Contig 3 (BSF2)	3	38.3	NC029802	nt (68.7) aa (76.7)	Segment S (RdRp): 748 nt- partial coding sequence	None	None
Porcine picobirnavirus (Segment S)- Contig 1 (BSF3)	1	43.2	NC029802	nt (68.5) aa (76.8)	Segment S (RdRp): 663 nt- partial coding sequence	None	None
Porcine picobirnavirus (Segment S)- Contig 2 (BSF3)	1	40.3	NC029802	nt (70.8) aa (76.0)	Segment S (RdRp): 697 nt- partial coding sequence	None	None
Green monkey picobirnavirus (BSF2)	5	84.9	NC034452	nt (65.1) aa (63.7)	RdRp: 1449 nt- partial coding sequence	None	R (518), R (519), E (520), R (538)

Green monkey picobirnavirus-Contig 1 (BSF3)	2	63.9	NC034452	nt (66.4) aa (65.1)	RdRp: 1091 nt- partial coding sequence	None	A (13), R (14), K (15), F (16)
Green monkey picobirnavirus-Contig 2 (BSF3)	2	61.5	NC034452	nt (65.0) aa (64.2)	RdRp: 1050 nt- partial coding sequence	None	T (294), P (295), S (331)
Green monkey picobirnavirus-Contig 3 (BSF3)	2	32.2	NC034452	nt (65.6) aa (63.6)	RdRp: 550 nt- partial coding sequence	None	None
Human picobirnavirus (Segment 2)-(BSF2)	9	64.9	NC007027	nt (63.8) aa (66.7)	Segment 2 (RdRp): 1133 nt- partial coding sequence	None	None
Human picobirnavirus (Segment 2)-Contig 1 (BSF3)	1	79.9	NC007027	nt (56.9) aa (57.3)	Segment 2 (RdRp): 1395 nt- partial coding sequence	ETNKEKL (41), DKRGVVAIQ (170), D (286), A (314)	W (8), Y (146), I (158), P (159), C (160), A (307)
Human picobirnavirus (Segment 2)-Contig 2 (BSF3)	2	68.7	NC007027	nt (67.1) aa (69.2)	Segment 2 (RdRp): 1199 nt- partial coding sequence	V (288)	None
Human picobirnavirus (Segment 2)-Contig 3 (BSF3)	1	32.7	NC007027	nt (66.2) aa (70.2)	Segment 2 (RdRp): 571 nt- partial coding sequence	T (292)	None
**Family *Picornaviridae***							
Porcine kobuvirus (BSF1)	6	25.8	NC016769	nt (89.7) aa (96.3)	2117 nt (partial coding sequence for polyprotein)	None	T (407), Q (408), D (409)
Porcine kobuvirus (BSF2)	1	98.9	NC011829	nt (89.1) aa (96.6)	8123 nt (complete coding sequence for polyprotein)	None	None
Porcine kobuvirus (BSF3)	4	33.0	NC011829	nt (90.6) aa (96.9)	2706 nt (partial coding sequence for polyprotein)	None	None
Porcine kobuvirus (BSF3)	6	54.8	NC016769	nt (88.0) aa (94.5)	4501 nt (partial coding sequence for polyprotein)	None	None

Pasivirus A (BSF2)	1	99.4	NC018226	nt (81.4) aa (88.8)	6876 nt (complete coding sequence)	EG (688)	T (350)
Pasivirus A (BSF3)	1	96.3	NC018226	nt (77.1) aa (81.7)	6672 nt (near complete coding sequence)	HG (688)	P (354)
Enterovirus 9 (BSF2)	1	99.4	NC004441	nt (79.6) aa (89.3)	7351 nt (complete coding sequence for polyprotein)	None	None
Enterovirus 9 (BSF3)	27	87.8	NC004441	nt (77.7) aa (88.2)	6485 nt (partial coding sequence for polyprotein)	None	None
Enterovirus goat (BSF2)	4	31.6	NC034267	nt (78.0) aa (90.0)	2354 nt (partial coding sequence for polyprotein)	None	T (496), G (497)
Enterovirus goat (BSF3)	4	37.2	NC034267	nt (73.4) aa (82.2)	2779 nt (partial coding sequence for polyprotein)	None	None
Sichuan takin enterovirus (BSF2)	5	32.3	NC037654	nt (75.0) aa (84.0)	2104 nt (partial coding sequence for polyprotein)	None	None
Sichuan takin enterovirus (BSF3)	5	43.5	NC037654	nt (74.0) aa (83.2)	2838 nt (partial coding sequence for polyprotein)	F (837)	E (500), Y (501)
Teschovirus A-Contig 1 (BSF2)	1	98.3	NC003985	nt (83.0) aa (89.7)	7001 nt (complete coding sequence for polyprotein)	P (332)	Q (956)
Teschovirus A-Contig 2 (BSF2)	3	92.0	NC003985	nt (80.8) aa (88.4)	6550 nt (partial coding sequence for polyprotein)	V (328), GT (654), LK (919)	None
Teschovirus A-(BSF3)	5	99.4	NC003985	nt (82.5) aa (90.2)	7078 nt (partial coding sequence for polyprotein)	D (333), M (919)	None
Sapelovirus 1 (BSF2)	1	99.7	NC003987	nt (84.4) aa (93.2)	7473 nt (complete coding sequence for polyprotein)	GSTA (132), ASAEQLGPFYPAI (892), A (2081)	None
Sapelovirus 1 (BSF3)	1	99.3	NC003987	nt (85.4) aa (94.9)	7438 nt (complete coding sequence for polyprotein)	STAG (133), A (2081)	None
Tottorivirus A (BSF2)	1	95.5	NC_055159	nt (62.5) aa (56.6)	6746 nt (partial coding sequence for polyprotein)	T (433), NV (491), P (498), L (636), Y (679), PG (708), PPAAT (781), S (862), LK (938), ISK (960), S (1003), APR (1353)	N (4), M (5), E (6), S (548), A (557), D (567), T (623), GCP (744), V (784), P (785), V (804), V (817), R (821), P (822), K (913), D (954), F (955), Y (973), G (981), K (1718)
**Order *Picornavirales***							

Posavirus 1 (BSF1)	2	96.1	NC023637	nt (94.8) aa (95.6)	9453 nt (partial coding sequence for polyprotein)	None	S (2610), S (2611), E (2612), K (2613), V (2614), N (2615), G (2616), N (2617), Y (2618), V (2619)
Posavirus 1 (BSF2)	1	99.3	NC023637	nt (88.6) aa (89.2)	9789 nt (complete coding sequence for polyprotein)	G (2428)	N (2906)
Posavirus 1 (BSF3)	1	99.3	NC023637	nt (88.6) aa (89.1)	9782 nt (complete coding sequence for polyprotein)	G (2428)	N (2906)
Posavirus 3 (BSF1)	8	24.1	NC028240	nt (80.8) aa (85.8)	2138 nt (partial coding sequence for polyprotein)	None	None
Posavirus 3 (BSF2)	7	21.4	NC028240	nt (80.2) aa (86.6)	1906 nt (partial coding sequence for polyprotein)	None	None
Posavirus 3 (BSF3)	1	99.6	NC028240	nt (81.5) aa (86.9)	8845 nt (near complete coding sequence for polyprotein)	PD (178), M (1107)	S (155), P (156), A (157), S (158), E (159)
Picornavirales Bu-1 (BSF2)	3	96.2	NC030745	nt (87.2) aa (89.9)	8877 nt (partial coding sequence for polyprotein)	H (1160), T (2577)	Q (91)
Picornavirales Bu-1 (BSF3)	3	48.8	NC030745	nt (87.4) aa (90.6)	4499 nt (partial coding sequence for polyprotein)	N (1160)	None
Picornavirales Tottori-HG1 (BSF3)	1	99.2	NC030744	nt (92.0) aa (95.6)	9787 nt (complete coding sequence for polyprotein)	None	None
**Family *Caliciviridae***							
Porcine enteric sapovirus (BSF2)	1	99.5	NC000940	nt (76.8) aa (85.0)	7290 nt (near complete coding sequence for polyprotein and complete coding sequence for small basic protein)	**Polyprotein:** PN (30), R (50)	**Polyprotein:** L (41)
**Family *Phenuiviridae***							
Dipteran hudivirus (Segment 3)-BSF1	2	47.9	NC032279	nt (70.4) aa (73.1)	592 nt (partial coding sequence for putative nucleoprotein)	None	None
Rice stripe tenuivirus (BSF3)	2	32.9	NC003753	nt (73.6) aa (83.5)	709 nt (partial coding sequence)	None	None
**Family *Dicistroviridae***							
Goose dicistrovirus (BSF2)	9	39.8	NC029052	nt (83.9) aa (87.5)	3636 nt (partial coding sequences for nonstructural polyprotein and capsid protein precursor)	**Capsid protein precursor:** SSSVK (520)	**Capsid protein precursor:** T (535), A (536)

Aphid lethal paralysis virus (BSF2)	7	20.5	NC004365	nt (97.7) aa (98.0)	2007 nt (partial coding sequences for nonstructural polyprotein and capsid protein precursor)	None	None
**Family *Partitiviridae***							
Aspergillus fumigatus partitivirus 2 (BSF2)	3	55.6	NC040757	nt (77.7) aa (83.7)	Segment 1 (RdRp): 1013 nt (partial coding sequence)	None	D (72), R (73), K (74), G (420)
Penicillium stoloniferum virus S (BSF2)	1	20.3	NC005976	nt (72.2) aa (70.1)	Segment 1 (RdRp): 356 nt (partial coding sequence)	None	None
**Family *Tobaniviridae***							
Porcine torovirus (BSF3)	24	33.7	NC022787	nt (87.4) aa (87.1)	9528 nt (partial coding sequence)	None	None
**Family *Retroviridae***							
Koala retrovirus (BSF3)	2	22.2	NC039228	nt (69.8) aa (77.6)	1869 nt (partial coding sequence for gag and polymerase proteins)	**Polymerase protein:** E (595)	None
**Family *Virgaviridae***							
Pepper mild mottle virus (BSF1)	1	98.0	NC003630	nt (99.7) aa (99.8)	6233 nt (near complete coding sequence)	None	None
Pepper mild mottle virus (BSF2)	7	88.6	NC003630	nt (99.6) aa (99.5)	5632 nt (near complete coding sequence)	None	None
Tobacco mild green mosaic virus (BSF1)	5	80.4	NC001556	nt (98.2) aa (98.3)	5108 nt (partial coding sequence)	gp1: P (1332)	None
Tobacco mild green mosaic virus (BSF2)	3	17.1	NC001556	nt (96.6) aa (96.0)	1084 nt (partial coding sequence)	None	None
**Family *Tombusviridae***							
Johnsongrass chlorotic stripe mosaic virus (BSF1)	3	93.0	NC005287	nt (75.5) aa (76.9)	4112 nt (partial coding sequence)	**Coat protein:** VG (24), AG (41), G (274)	**Coat protein:** N (37)
**Unclassified RNA viruses**							

Beihai tombus-like virus 15 (BSF2)	3	40.9	NC033202	nt (58.1) aa (54.5)	1361 nt (partial coding sequence)	**Hypothetical protein 1:** LSQ (538)	**Hypothetical protein 1:** N (525), D (526), E (531)
Thika virus (BSF2)	8	39.6	NC027127	nt (65.8) aa (67.2)	3603 nt (partial coding sequence)	L (2119)	P (2090), G (2091)
Hubei picorna-like virus 15 (BSF2)	11	39.6	NC032757	nt (90.2) aa (94.2)	3926 nt (partial coding sequence)	None	None
Hubei tombus-like virus 17 (BSF3)	3	49.9	NC032813	nt (63.4) aa (61.6)	1854 nt (partial coding sequence)	None	**Hypothetical protein 2:** T (98) **Hypothetical protein 3:** Q (233)
Wuhan insect virus 23 (BSF3)	1	26.2	NC032146	nt (69.5) aa (73.6)	387 nt (partial coding sequence)	None	None

BSF = Backyard swine farm; nt = nucleotides; aa = amino acids.
